# BGFit: management and automated fitting of biological growth curves

**DOI:** 10.1186/1471-2105-14-283

**Published:** 2013-09-25

**Authors:** André Veríssimo, Laura Paixão, Ana Rute Neves, Susana Vinga

**Affiliations:** 1 INESC-ID, R Alves Redol 9, 1000-029 Lisbon, Portugal; 2 Instituto de Tecnologia Química e Biológica, Universidade Nova de Lisboa (ITQB-UNL), Avenida da República, 2780-157 Oeiras, Portugal; 3 Centre for Intelligent Systems, IDMEC-LAETA, IST-UL, Av. Rovisco Pais 1, 1049-001 Lisbon, Portugal; 4 Present address: CED-Discovery, Chr Hansen A/S, 10-12 Bøge Allé, DK2970, Hørsholm, Denmark

## Abstract

**Background:**

Existing tools to model cell growth curves do not offer a flexible integrative approach to manage large datasets and automatically estimate parameters. Due to the increase of experimental time-series from microbiology and oncology, the need for a software that allows researchers to easily organize experimental data and simultaneously extract relevant parameters in an efficient way is crucial.

**Results:**

BGFit provides a web-based unified platform, where a rich set of dynamic models can be fitted to experimental time-series data, further allowing to efficiently manage the results in a structured and hierarchical way. The data managing system allows to organize projects, experiments and measurements data and also to define teams with different editing and viewing permission. Several dynamic and algebraic models are already implemented, such as polynomial regression, Gompertz, Baranyi, Logistic and Live Cell Fraction models and the user can add easily new models thus expanding current ones.

**Conclusions:**

BGFit allows users to easily manage their data and models in an integrated way, even if they are not familiar with databases or existing computational tools for parameter estimation. BGFit is designed with a flexible architecture that focus on extensibility and leverages free software with existing tools and methods, allowing to compare and evaluate different data modeling techniques. The application is described in the context of bacterial and tumor cells growth data fitting, but it is also applicable to any type of two-dimensional data, e.g. physical chemistry and macroeconomic time series, being fully scalable to high number of projects, data and model complexity.

## Background

Modeling cell growth and estimating curve parameters from data is a common task in areas ranging from microbiology to oncology.

In microbiology studies, maximal growth rate and maximal biomass are probably the two best studied bacterial growth properties. In a nutshell, these physiological properties provide a rough reflection of how well a bacterium cell benefits from a particular set of nutrients. Thus, they can be used to guide a myriad of applications. One such example, is the utilization of growth rate maximization as an objective in constraint-based reconstruction and analysis of metabolic networks.

In oncology, it is also crucial to model tumors growth and understand their dynamics under different internal and external perturbations. For example, linking growth parameters with pharmacokinetic-pharmacodynamic (PK/PD) models can help predicting the responses of tumor dynamics when exposed to distinct drug regimes [1]. This knowledge can be further used to optimize the design of new experiments and support preclinical development of oncology drugs.

Hence, it is relevant for experimentalists to extract these key parameters from curves in order to characterize cell and tissue physiology, such as maximum growth rates, lag phase and asymptotic maximum OD. Furthermore, it is also interesting to be able to compare fittings obtained with different models. Due to the development of high-throughput techniques, the amount of data being generated is growing fast, hindering their management in large collaborative projects and also hampering model identification procedures.

Several dynamic models based on differential and algebraic equations have already been proposed and are extensively used in these fields. These include sigmoidlike curves such as Logistic, Gompertz, Richards, Schnute and Stannard [2,3]. The fitting of these curves to growth data is usually performed using in-house software or freely available tools. These include DMFit (available at http://www.ifr.ac.uk/safety/dmfit/) and GInaFiT [4], which offer an Excel add-in to model data according to several implemented dynamic models, along with packages provided for software R, namely *grofit*[5] and *cellGrowth* in Bioconductor. Other tools such as MicroHibr (http://www.microhibro.com/) are available as a web-application but with limited functionality. Recently developed databases such as ComBase [6] and LabBase [7] try to aggregate and store time-series of bacterial growth under several experimental conditions, serving as benchmarks for Predictive Microbiology.

Although dynamic models and databases for biological growth data have now reached a mature state, there is still no easy-to-use software, to our knowledge, that allows experimentalists not familiar with computational tools to extract relevant parameters in an easy and automated way and simultaneously efficiently manage their data. This constitutes the main motivation for the development of BGFit, which further allows to integrate more sophisticated and complex models, both algebraic and differential, due to its flexible and expandable architecture. Its main utility is thus to provide a user-friendly web-service that couples database management with model inference, with expected applicability in several areas of research. The examples here provided (data available for illustrative purposes in the webpage) include Microbiology projects, with the estimation of bacterial growth curves under different sugars, and Oncology, where models for the time evolution of carcinoma weight are inferred.

## Implementation

BGFit web-application serves both as: (i) an automated fitting tool for experimental data using an extensible set of dynamic models through a distributed architecture and; (ii) a data repository that stores and manages experimental data.

The data modeling features allow users to choose a dynamic model and estimate the parameters that best describe the dataset. With this information BGFit simulates the estimated curve and presents the results in a chart along with the original dataset and goodness-of-fit measures.

This automated process can be applied to single dataset, or to a collection that aggregates similar or complementary data, such as replicates of an experiment. This provides both a global view on aggregated data and a fine control on specific measurements.

BGFit’s repository of dynamic models allow users to apply their own models, as well as take advantage of an existing and expandable set of contributed models, each bestowing to a richer environment. With this functionality it is possible to compare the results of different fittings in a single dashboard. The models currently implemented are Baranyi, Gompertz, Logistic and Richards models [2,8], first and second order polynomial regression, exponential decay, Lumry-Eyring - LENP type Ib (ODE) [9] for modeling the kinetics of irreversible protein aggregation, Hyperbolastic growth model of type III (H3) [10] and Live Cell Fraction model [11,12]. To complement the dynamic modeling feature, users can also apply manual regression on the data, traditionally performed as a linear fitting in logarithmic scale.

While not intended to be exhaustive, this list implements a wide set of algebraic and differential models that are used in many areas or research and serves as a support for future expansions by users.

The data-management features supports the modeling process and facilitates the collaboration by creating a central point of access. One of the motivations for this application is the need to have a better workflow for collaboration, avoiding the exchange of files using traditional methods, such as emails and shared folders. Thus, BGFit features a hiearchical-based data storage where users can define their own teams and attribute read/write permissions accordingly. Additionally the public scope can also be defined, allowing to openly share and publish the data online.

All the input data and results, such as the time series, estimated parameters, model simulations and charts, are available for direct download to further analysis.

The entire source code for BGFit and the implemented models are available online, as well as the instruction to setup a fully functional installation locally. This addresses data confidentiality by allowing each laboratory to keep a local BGFit version for private projects.

### Architecture and data structure

BGFit is developed using open-source frameworks and free libraries allowing for a high degree of flexibility and creating a modular system constituted by Ruby on Rails, MySQL, Octave, MathJax and Google Chart Tools.

The application is designed using a model-view-controller architecture effectively separating data-management and dynamic modeling that is performed using extensions that are decoupled from the web-application.

The modelling extensions only require the implementation of the necessary interface and for it to be deployed on a location that is accessible by BGFit. This approach allows for every component of BGFit to be deployed online, encouraging collaboration and the reutilization of these tools. It can also be used in a local installation while keeping the access to all the developed models.

**Figure 1:**
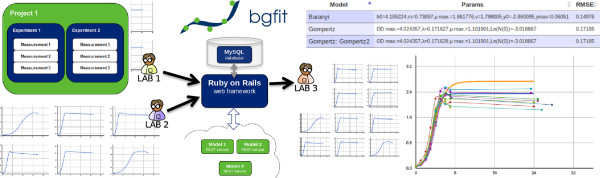
**BGFit architecture.** BGFit is designed using a model-view-controller architecture and the data is organized hierarchically into projects, experiments and measurements. Users can introduce their data through the web-based platform, while other collaborators independently fit any implemented model. BGFit was developed using Ruby on Rails web-framework, MySQL, Octave/MATLAB and Google Chart Tools.

Input data is stored using a hierarchical-based organization with three different layers. The top-level layer, *project*, defines global properties for the project, such as user permissions and whether it is publicly available. The middle layer, *experiments*, aggregates the different results in folders. The bottom layer, *measurements*, is the user’s actual data and can store 3-dimensional annotated data, although only the first two dimensions are used in the modeling extensions for now (Figure 1). BGFit represents a central repository for data, models and fittings.

### Modelling extensions

One of the strengths of BGFit is that it allows to easily expand the dynamic models. Modeling the data in the application is performed through a REpresentational State Transfer (REST) web-service that receives a set of parameters as input and returns the function’s result.

The web-service should support two functions and a baseline for comparisons between different models, e.g., root mean square error (RMSE): 1) Parameter estimation - which takes the data-points, such as time series, a range set for the parameters and outputs the estimated parameters using linear/nonlinear regression and 2) Model simulation - that receives a set of parameters for the model as input and returns a simulated curve.

The modeling extension should implement these functions to be fully compliant. This approach forces a strict interface for communication, but on the other hand, it offers flexibility on the implementation of the model as it is technological agnostic.

The necessary technical documents, templates and examples are fully described in the Model Blackbox public repository (https://github.com/averissimo/model_blackbox), providing a starting point for users to create and implement their own interface-compliant models.

The available templates offer two approaches implemented in Octave and Matlab’s numerical computing environments, either as a script for Octave, making it possible to deploy the modeling extensions without any licensing issues, or as a standalone application for Matlab, taking advantage of SBToolbox2 [13] functions.

## Results and discussion

In order to illustrate the organization of the data and how to retrieve the available information, we will exemplify the application of BGFit tool in two different projects: 1) bacterial growth fitting and 2) tumor cell growth. The data is available at the webpage, along with all the necessary documentation. (see also Additional file 1)

In Table 1 some of the models implemented in BGFit are shown.

**Table 1:** Some models implemented in BGFit

**Name**	**Equation**	**Reference**
Hyperbolastic growth type III (H3)	$$\begin{array}{l} P(t)\;=\;M\;-\;\alpha \;\text{exp}\;(-\delta t^{\gamma }\;-\;\text{arcsinh}(\theta t)),\\ \;\alpha \;=\;(M\;-\;P_{0})\;\text{exp}(\delta t_{0}^{\gamma }+\;\text{arcsinh}\;(\theta t_{0})) \end{array}$$	[10]
Live Cell Fraction	$\frac{\text{dr}}{\text{dt}}=r(t)/3\cdot ((\alpha +\delta )\cdot 3\cdot \lambda /(3\cdot \lambda +r(t))-\delta )$	[11,12]
Baranyi	$$\begin{array}{l} y(t)=y_{0}+\mu_{\text{max}}t+\frac{1}{\mu_{\text{max}}}\ln\left(e^{-v\cdot t}+e^{-h_{0}}-e^{-v\cdot t-h_{0}}\right)\\ \;-\frac{1}{m}\ln\left(1+\frac{e^{m\cdot \mu_{\text{max}}t+\frac{1}{\mu_{\text{max}}}\ln\left(e^{-v\cdot t}+e^{-h_{0}-e^{-v\cdot t-h_{0}}}\right)-1}}{e^{m(y_{\text{max}}-y_{0})}}\right) \end{array}$$	[8]
		
Gompertz	$y(t)\;=\;A\;\cdot \;\text{exp}\;\left\{-\text{exp}\;\left[\frac{\mu_{\text{max}}\cdot e}{A}\left(\lambda -t\right)+1\right],\;\right\}$	[2]

**Figure 2:**
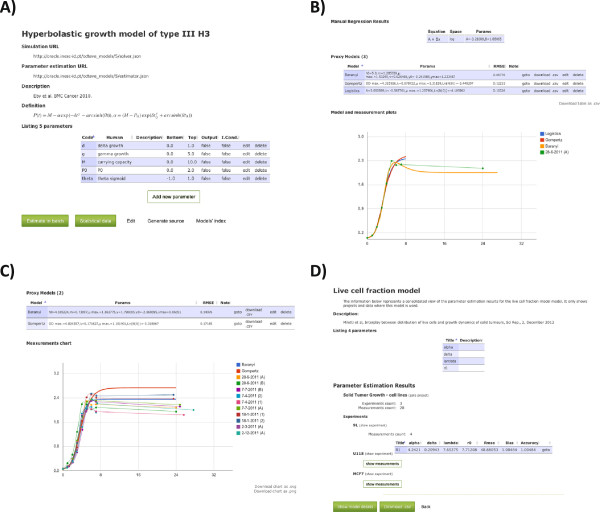
**BGFit modeling examples.** Screenshoots of BGFit application, illustrating different features of the tool. **A)** Model definition – example on the Hyperbolastic growth model of type III (H3) description. **B)** Single measurement fitting – comparison between several models for the same time-series growth data on bacterial growth curves. **C)** Aggregated experiment fitting – illustrated the simultaneous estimation of several measurements or replicates of the same experiment. **D)** Aggregated estimation results – shows the model page where the user can assess and download all the information for the estimated projects.

Figure 2 illustrates several features of BGFit. Panel A) represents a specific model overview webpage (Hyperbolastic growth), where the user can define the algebraic or differential equation, along with all the detailed description regarding the parameters, such as the expected search range values and which of them represent initial conditions. The source code for estimation and simulation is automatically generated, thus expanding the model collection currently available. In this page the user can also download all the statistical data of the fittings performed. Panel B) shows one measurement fitted with different models, including a manual regression, allowing to compare them graphically and numerically. The simulations are plotted along with the original experimental data, further supporting visual inspection of the results. Panel C) illustrates the simultaneous estimation of different measurements of the same experiment. This allows to fit a model to several replicates, useful for finding an average model for similar experimental conditions. Panel D) shows all the estimation results obtained for a given model, allowing the user to download the information in a CSV file.

The results obtained by BGFit are in good agreement with previous estimation procedures and are made directly available at the webpage for further analysis.

In particular, we have compared our results with published data and also with R software package *grofit*[5], to demonstrate the consistency of the obtained fittings.

More specifically, we have used as a first testing set the time evolution of the weight of Solid Ehrlich Carcinoma treated with combined IAA and DMSO described in [10]. These data were fitted with a hyperbolastic growth model of type III (H3), proposed by Eby and colleagues. Regarding this comparison, BGFit returned similar results. The parameters reported in the original paper were (*M*=7.533, *δ*=3.594·10^−9^, *γ*=4.712 and *θ*=0.004, RMSE=0.07264) and those obtained by BGFit were (*M*=7.547, *δ*=5.103·10^−9^, *γ*=4.630 and *θ*=0.004, RMSE=0.066), showing an excellent agreement. BGFit estimation slightly improved the previous fitting, related with a better adjustment of the curve in the initial phase (see figures at http://kdbio.inesc-id.pt/bgfit/experiments/113/measurements/270for details).

The second comparison performed was with the results obtained by state-of-the-art *grofit* package in R [5]. We have used the testing datasets provided upon installation and compared BGFit with *grofit* results on Gompertz and Richards models (see all seven measurements at http://kdbio.inesc-id.pt/bgfit/projects/62/experiments/146, along with figures and tables), thus demonstrating the consistency of our results.

The obtained fittings and corresponding comparisons and simulation results are fully available at the webpage under projects *Tumor Growth* and *Comparing BGFit with R* respectively.

## Conclusions

The main contributions of BGFit are delivering a platform for automated data modeling of large time-series dataset and providing a baseline for comparison between different models, either novel or already described in the literature.

By designing the application based on a distributed architecture that separates heavy calculations from the data management and repository, computational load is distributed through different locations and network of models. This allows BGFit to scale as the userbase grows.

BGFit supports collaborative projects by providing a central repository which can be used by several teams simultaneously, handling large experimental datasets through a clean and hierarchy-based organization of the data. BGFit allows users to implement and reuse an ever growing network of models, to improve validation of their methods, thus supporting model comparison and selection procedures.

BGFit is designed as a parameter estimation platform for any type of two-dimensional data. Despite being described in the context of cell growth data, the application can easily be used in other areas with different dynamic and algebraic models, such as physical chemistry and econometrics.

## Availability and requirements

**Project name:** BGFit

**Project home page: **http://kdbio.inesc-id.pt/bgfit

**Operating system(s):** Platform independent

**Programming language:** Ruby

**Other requirements:** Ruby 1.9.3 or higher

**License:** GNU GPL v2

**Any restrictions to use by non-academics:** Only those imposed already by the license.

## Competing interests

The authors declare that they have no competing interests.

## Authors’ contributions

AV implemented and designed the application, LP and ARN provided experimental data and supported the analysis of the results, SV designed the application, analyzed the results and organized manuscript writing. All authors read and approved the final manuscript.

## Supplementary Material

Additional file 1 **BGFit complete documentation.** BGFit application full user and technical documentation as in April 2013 (for reference purposes). The most recent version is available at http://kdbio.inesc-id.pt/bgfit/bgfit_documentation.pdf Click here for file

## References

[B1] Simeoni M Magni P Cammia C De Nicolao G Croci V Pesenti E Germani M Poggesi I Rocchetti M Predictive Pharmacokinetic-Pharmacodynamic modeling of tumor growth kinetics in xenograft models after administration of anticancer agents Cancer Res 2004 64 3 1094 1101 10.1158/0008-5472.CAN-03-2524 14871843

[B2] Zwietering MH Jongenburger I Rombouts FM van’t Riet K Modeling of the bacterial growth curve Appl Environ Microbiol 1990 56 6 1875 1881 16348228 10.1128/aem.56.6.1875-1881.1990 PMC184525

[B3] Peleg M Corradini MG Microbial growth curves: what the models tell us and what they cannot Crit Rev In Food Sci And Nutr 2011 51 10 917 945 10.1080/10408398.2011.570463 21955092

[B4] Geeraerd A Valdramidis V Van Impe J GInaFiT, a freeware tool to assess non-log-linear microbial survivor curves Int J Food Microbiol 2005 102 95 105 10.1016/j.ijfoodmicro.2004.11.038 15893399

[B5] Kahm M Hasenbrink G Lichtenberg-Frat’e H Ludwig J Kschischo M grofit: Fitting biological growth curves with R J Stat Softw 2010 33 7 1 21 [http://www.jstatsoft.org/v33/i07/]20808728

[B6] Baranyi J Tamplin ML ComBase: A common database on microbial responses to food environments J Food Prot 2004 67 9 1967 1971 15453591 10.4315/0362-028x-67.9.1967

[B7] Psomas AN Nychas GJ Haroutounian SA Skandamis P LabBase: development and validation of an innovative food microbial growth responses database Comp Electron Agric 2012 85 99 108

[B8] Baranyi J Roberts T McClure P A non-autonomous differential equation to model bacterial growth Food Microbiol 1993 10 1 43 59 10.1006/fmic.1993.1005

[B9] Roberts CJ Kinetics of irreversible protein aggregation: analysis of extended Lumry-Eyring models and implications for predicting protein shelf life J Phys Chem B 2003 107 5 1194 1207 10.1021/jp026827s

[B10] Eby WM Tabatabai MA Bursac Z Hyperbolastic modeling of tumor growth with a combined treatment of iodoacetate and dimethylsulphoxide BMC Cancer 2010 10 509 10.1186/1471-2407-10-509 20863400 PMC2955040

[B11] Chignola R Schenetti A Chiesa E Foroni R Sartoris S Brendolan A Tridente G Andrighetto G Liberati D Oscillating growth patterns of multicellular tumour spheroids Cell Prolif 1999 32 39 48 10.1046/j.1365-2184.1999.3210039.x 10371302 PMC6726320

[B12] Milotti E Vyshemirsky V Sega M Chignola R Interplay between distribution of live cells and growth dynamics of solid tumours Sci Rep 2012 2 990 doi: 10.1038/srep00990. Epub 2012 Dec 18 23251776 10.1038/srep00990 PMC3524520

[B13] Schmidt H Jirstrand M Systems biology toolbox for MATLAB: a computational platform for research in systems biology Bioinformatics 2005 22 4 514 515 16317076 10.1093/bioinformatics/bti799

