# Supplementary material for: BGFit: management and automated fitting of biological growth curves
Source: BMC Bioinformatics. 2013 Sep 25;14:283. doi: 10.1186/1471-2105-14-283 (PMC3848918; doi:10.1186/1471-2105-14-283)
Supplement: Additional file 1 — BGFit complete documentation. BGFit application full user and technical documentation as in April 2013 (for reference purposes). The most recent version is available at http://kdbio.inesc-id.pt/bgfit/bgfit_documentation.pdf [file 1471-2105-14-283-S1.pdf]

BGFit

INESC-ID

User and Technical Documentation

Supplementary material to reference to paper

Authors:
André Veríssimo
Susana Vinga

Contacts:
svinga@kdbio.inesc-id.pt

April 24, 2013

Contents

1 Introduction 3
1.1 Source Code 3
1.2 Availability 3

2 Architecture 3

3 Data organization 4

4 Tutorials on how to perform tasks in BGFit 6
4.1 Layout Description 6
4.2 Create a New Team 6
4.3 Adding an existing User to a Team 7
4.4 Sign Up with a New User 8
4.5 Login 8
4.6 Insert new data measurements 10
4.7 Estimate Parameters 13
4.8 Multiple Estimation 15
4.8.1 Aggregation 16
4.8.2 Batch estimation 17
4.9 Generate a new Model 19
4.9.1 Create a new Model 19
4.9.2 Add models parameters (if added later on the details page) 21
4.9.3 Generate source files 21
4.9.4 Download source files 22
4.10 Manual Regression 24

5 Modeling Extensions 27
5.1 Overview 27
5.2 Download 27
5.3 Technical Overview 27
5.4 Models 27
5.5 Requirements 28
5.5.1 Requirements for Octave-based models 28
5.5.2 Requirements for Matlab-based models 29
5.5.3 Requirements for SBTOOLBOX2-based models 29
5.6 Structure 29
5.7 Create a new model blackbox 29
5.7.1 Octave / Matlab model 29
5.7.2 Compile for octave 30
5.7.3 Compile for matlab 30
5.7.4 Compile for SBTOOLBOX2 model 30
5.8 Test the model 30
5.9 Deploy 31
5.9.1 Octave 31

5.9.2 Matlab or SBTOOLBOX2 31

6 Questions and Sugestions 32

List of Figures

1 Application's Architecture 4
2 Data structure 5
3 Layout of application 6

1 Introduction

Existing tools to model bacterial growth curves do not offer enough automated methods adequate for large datasets neither present a standard nor a flexible approach. BGFit provides a unified tool that offers a rich set of dynamic models for automatically estimate model parameters along with efficiently manage experimental time-series data in an structured way.

BGFit was designed with a flexible architecture that focus on extensibility and leverages free software with existing tools and methods. BGFit positions itself as a platform to compare and evaluate different data modeling techniques and extract relevant information from data.

The application is described in the context of bacterial growth data fitting, but it is also applicable to other types of two-dimensional data, e.g., from cancer growth experiments to macroeconomic data.

1.1 Source Code

- BGFit source code
- Model extension source code

All results are stored by the application and are downloadable by the user.

1.2 Availability

BGFit is currently available online at http://oracle.inesc-id.pt with all available functionality and several dynamic models that are further described at section 5.4.

The application is available for a guest user, that can browse and access detail on public data. However, to introduce and manage data or models it is required to register a user with a valid email.

2 Architecture

BGFit is developed using open-source frameworks and free libraries allowing for a high dregree of flexibility and creating a modular system. Ruby on Rails, MySQL, Octave, MathJax and Google Chart Tools are some examples.

The application is designed using a model-view-controller architecture effectively separating data-management and dynamic modeling that is performed using extensions that are decoupled from the web-application.

The modeling extensions only require the implementation of the necessary interface and for it to be deployed on a location that is accessible by BGFit. This approach allows for every component of BGFIt to be deployed online, encouraging collaboration and the reutilization of these tools. Nevertheless, it can also be used in a local installation.

The default modeling extension pack that describes different bacterial growth models (such as Baranyi, Gompertz, Logistics, etc. . .) is implemented in Octave/Matlab. These modeling extensions are also released with BGFit and are described in their documentation, as well as a template model from which all the implemented models derive. This provides a starting point for users to create their own models.

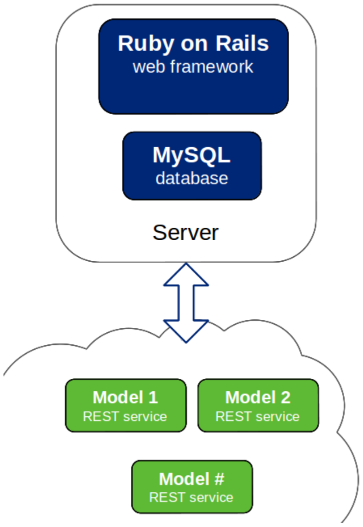

Figure 1: Application's Architecture

3 Data organization

Data management is divided in three layers: project (top-level folder) → experiment (folder) → measurement (actual data)

- Project: Top-level folder where permissions and other properties are set;
- Experiment: Folder to organize and aggregate data by typology;
- Measurement: Actual data, e.g. replicates.

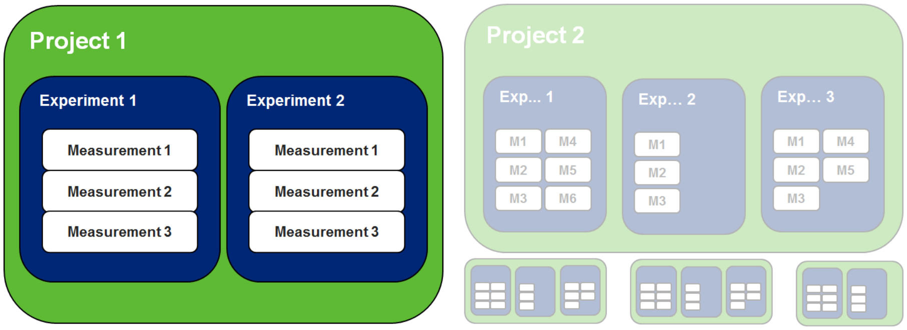

Figure 2: Data structure

4 Tutorials on how to perform tasks in BGFit

4.1 Layout Description

The application's layout is organized in three different areas (as shown in the image below):

1. Primary menu: Main navigation and contextual actions are available here;
2. User/Team management: Manage its account and the teams where he collaborates;
3. Content

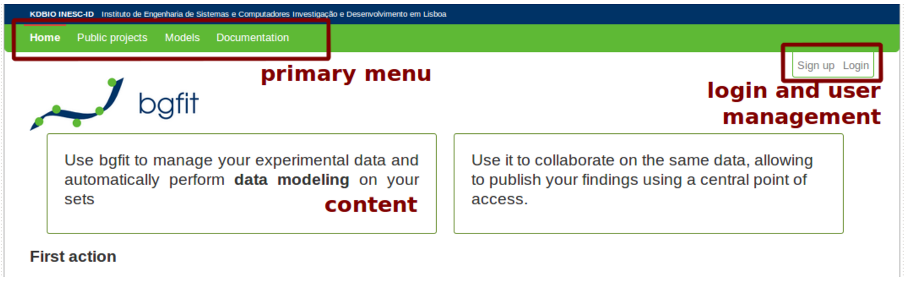

Figure 3: Layout of application

4.2 Create a New Team

BGFit allows for teams to collaborate on the project at hand, being able to work and share on the same data.

1. To create a new team a user needs to login to the application and click on the "My teams" link on to top right corner

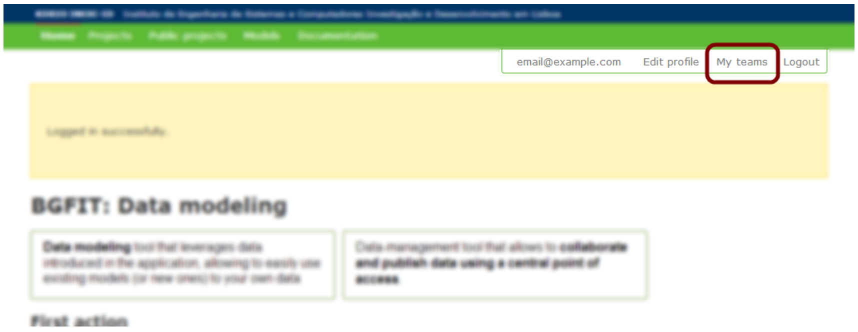

2. Click on "New team"

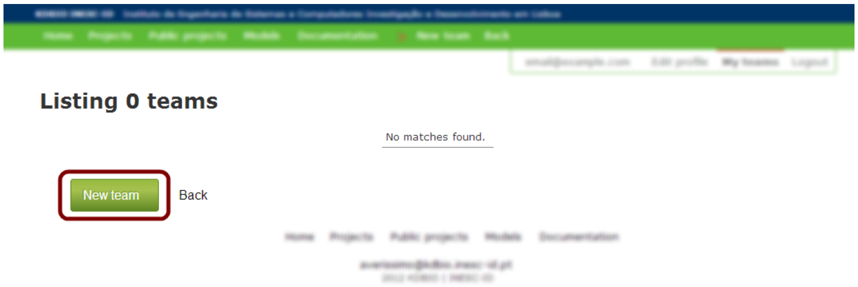

3. Select a name for the team

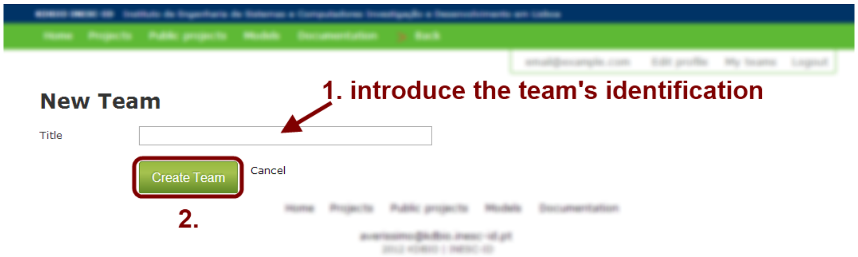

4.3 Adding an existing User to a Team

Goto the desired team and add a membership

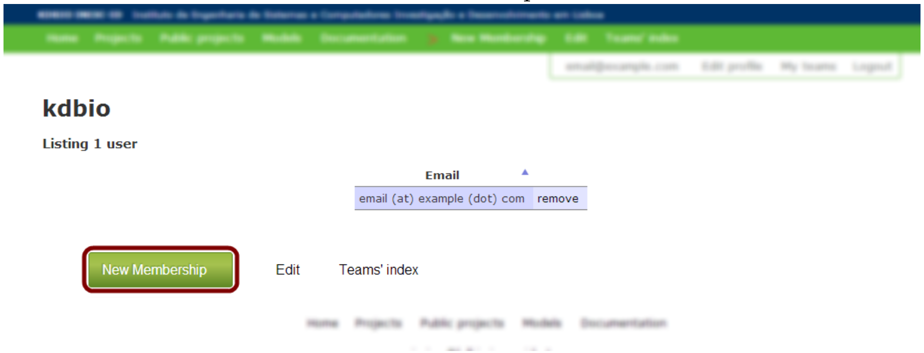

4.4 Sign Up with a New User

Any user can register a new user in BGFit, having to provide an email address and a password.

This process is available using the "User/Team management" menu on the top right corner.

1. Click on the "Sign up" link on the top right corner

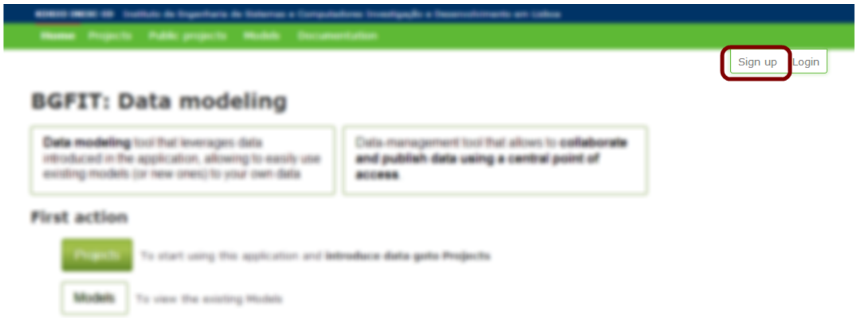

2. Introduce the required information

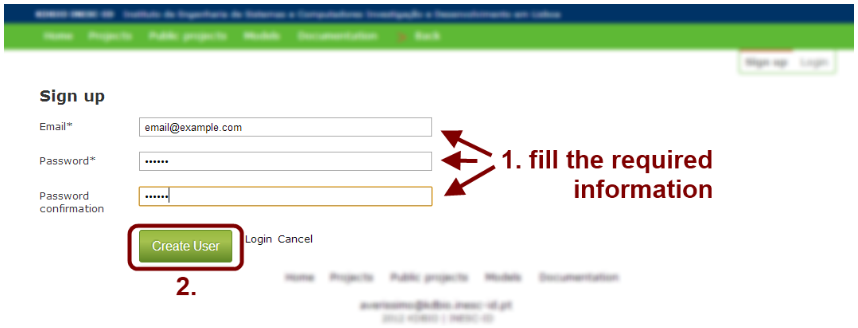

3. Success message

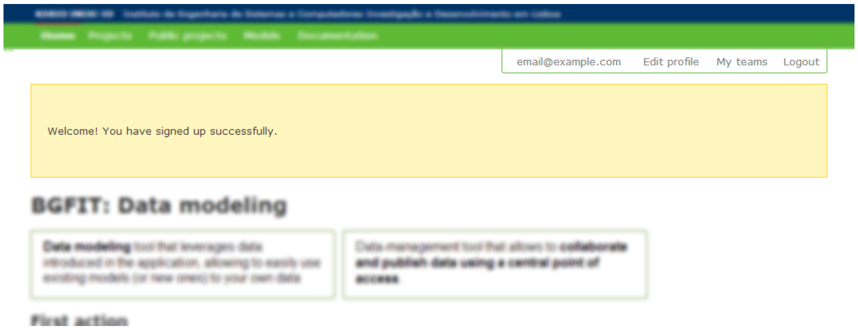

4.5 Login

1. Click on the "Login" link on the top right corner

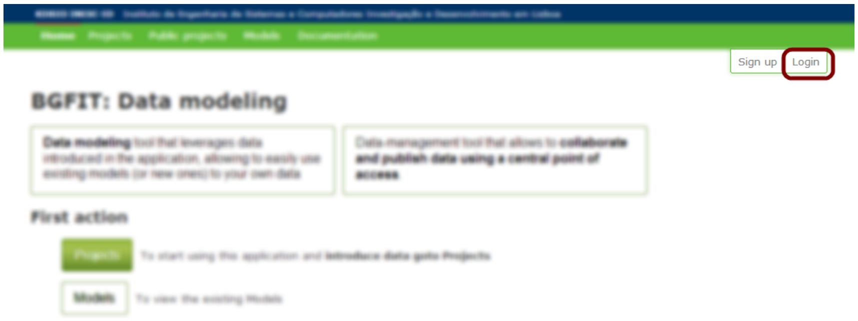

2. Introduce the login information

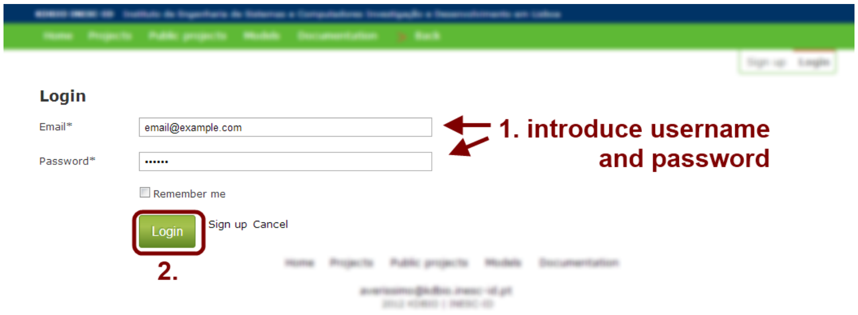

4.6 Insert new data measurements

1. Start by creating a new project

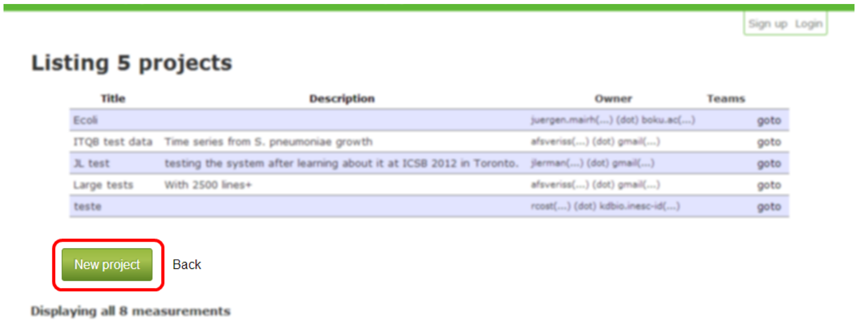

2. Fill information about the project

   Check the "published?" checkbox if the project should be public

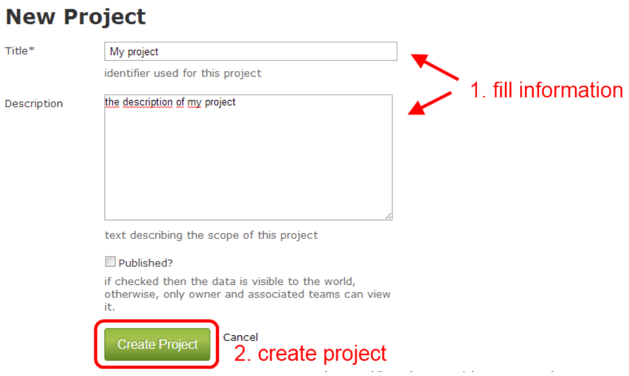

3. Insert data

   This can be accomplished in one of two ways:

   (a) Create a measurement directly using the default experiment folder

   (b) First create an experiment folder and then introduce measurements inside the folder

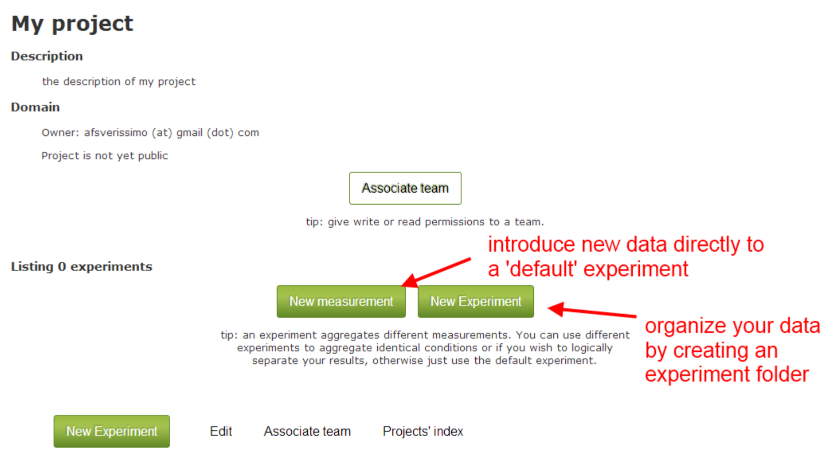

4. Filling data

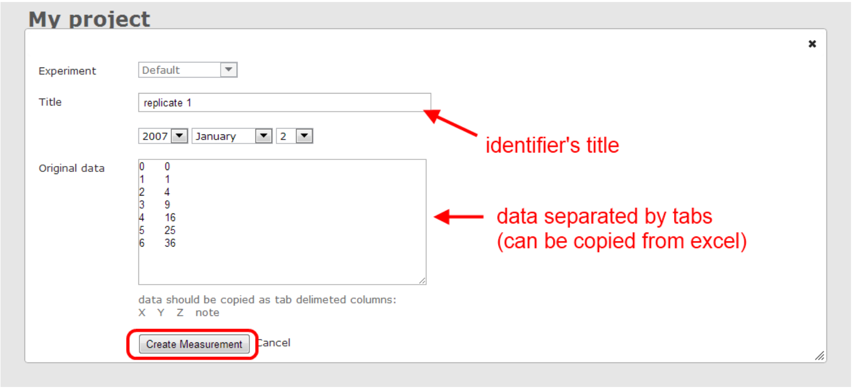

5. A view of the data showing the plot

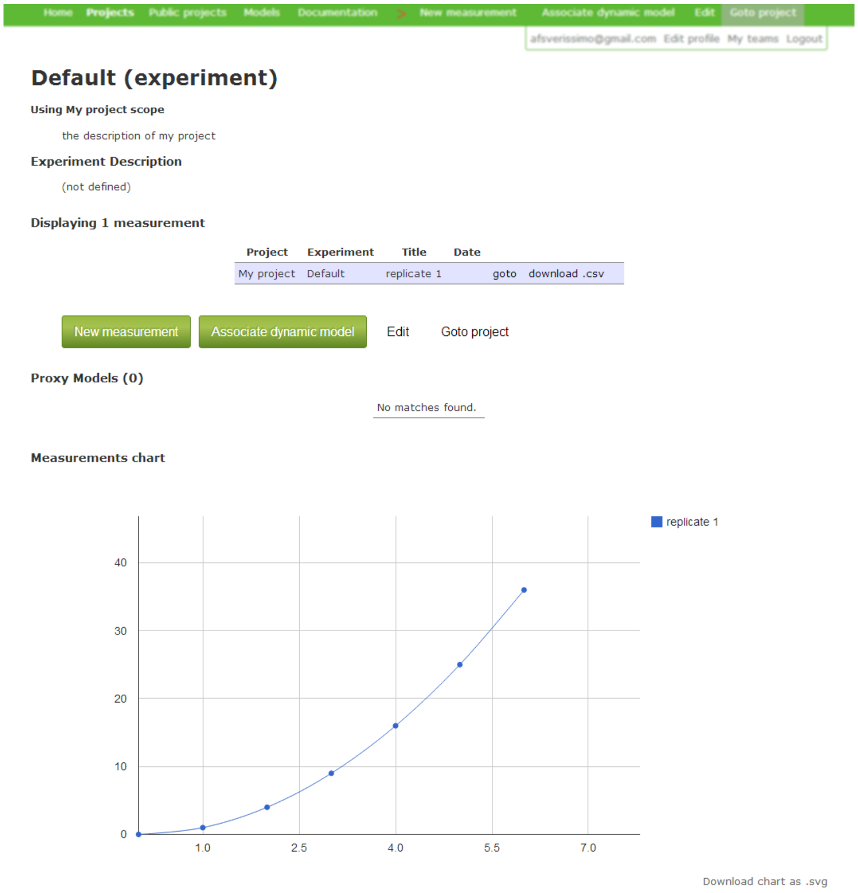
afsverissimo@gmail.com Edit profile My teams Logout
Default (experiment)
Using My project scope
the description of my project
Experiment Description
(not defined)
Displaying 1 measurement
Project Experiment Title Date
My project Default replicate 1 goto download .csv
New measurement
Associate dynamic model
Edit
Goto project
Proxy Models (0)
No matches found.
Measurements chart
replicate 1
40
30
20
10
0
1.0
2.5
4.0
5.5
7.0
Download chart as .svg

4.7 Estimate Parameters

1. Associate a model to measurement / experiment

   An user must first navigate to a measurement or experiment and click on"Associate model"

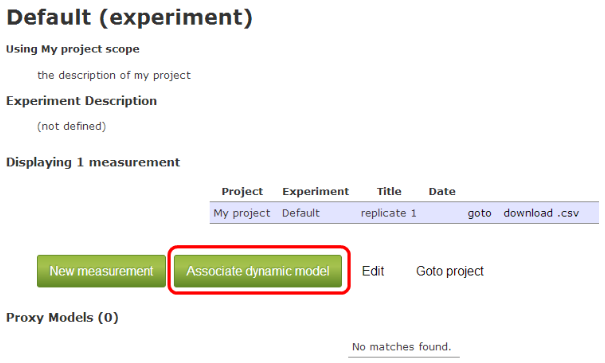

2. Choose from existing models

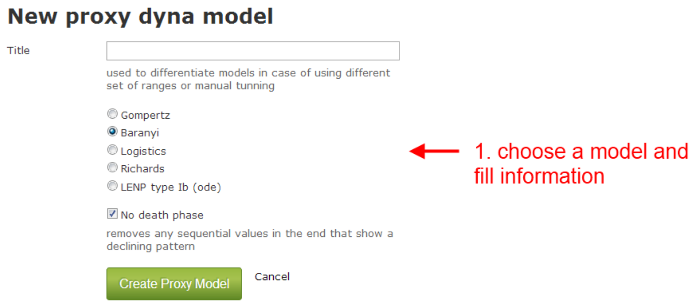

3. Calculate the parameters for the model

   Select a range for each of the parameters to better narrow the value each parameter can take. Or leave the default values that are taken from the model definition.

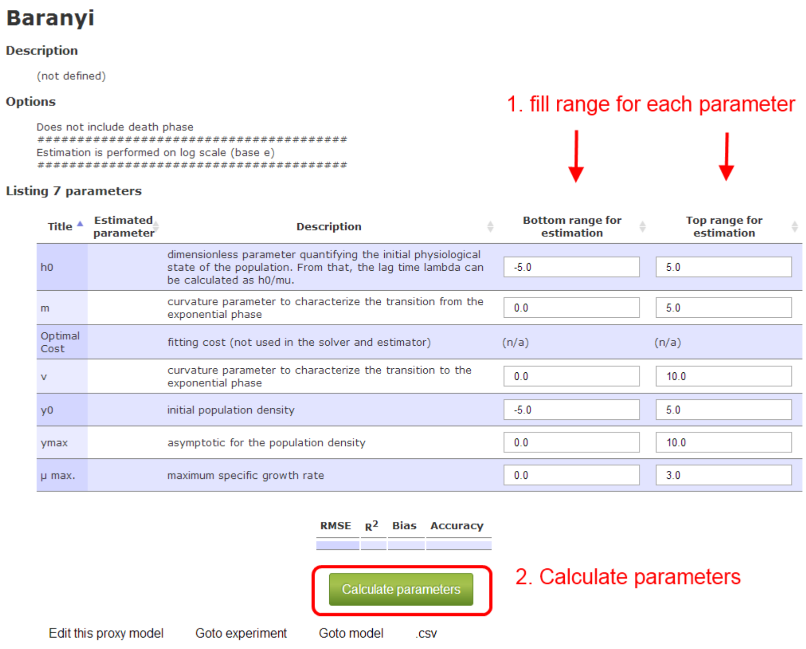

4. Results

   After successfully calculating the parameters the user is presented with the results: parameters values, statistical measures and a visual plot of the fitting.

   If the estimated parameters reaches the internal range, displayed as an highlighted shadow in red, a new range should be tested and a recalculation should be performed.

Baranyi

Laura S. pneumoniae | ITQB model's description for 18-1-2011 (2) measurement:

(not defined)

Description

(not defined)

Options

Does not include death phase
##
Estimation is performed on log scale (base e)
##

Listing 7 parameters

Title	Estimated parameter	Description	Bottom range for estimation	Top range for estimation
h0	**3.455956**	dimensionless parameter quantifying the initial physiological state of the population. From that, the lag time lambda can be calculated as h0/mu.	-5.0	5.0
m	**0.675574**	curvature parameter to characterize the transition from the exponential phase	0.0	5.0
Optimal Cost	**0.01313001196375**	fitting cost (not used in the solver and estimator)	(n/a)	(n/a)
v	**1.946531**	curvature parameter to characterize the transition to the exponential phase	0.0	10.0
y0	**-2.813356**	initial population density	-5.0	5.0
ymax	**0.0**	asymptotic for the population density	0.0	10.0
μ max.	**1.812065**	maximum specific growth rate	0.0	3.0

RMSE	R^2	Bias	Accuracy
0.08071	0.99347	1.00011	0.99989

Recalculate parameters

Edit this proxy model   Goto measurement   Goto model   .csv

Baranyi's Curve

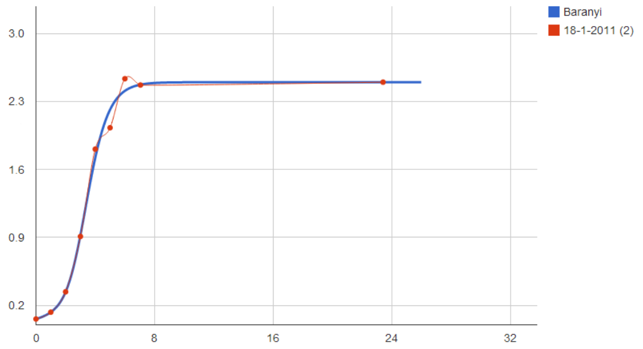

4.8 Multiple Estimation

There are two methods to perform multiple parameter estimation, one using different measurements to estimate parameters and the other to perform the parameter estimation on several measurements in parallel.

1. Aggregation: Perform parameter estimation on several measurements under an experiment;
2. Batch estimation: Performs parallel estimation to multiple measurements.

4.8.1 Aggregation

The process to perform parameter estimation using as input all experiment's measurements is identifcal to the process described above.

The difference lays in the context where the model association is performed. To aggregate the measurement the "Associate model" should be done in the experiment's detail page.

The proxy models will be editable and shown identically as above.

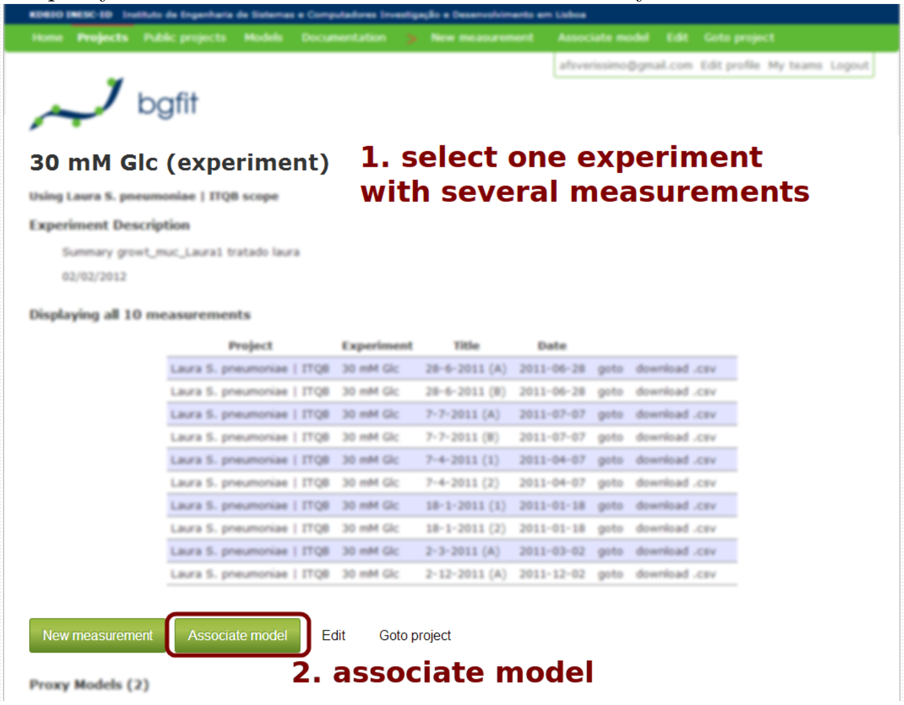

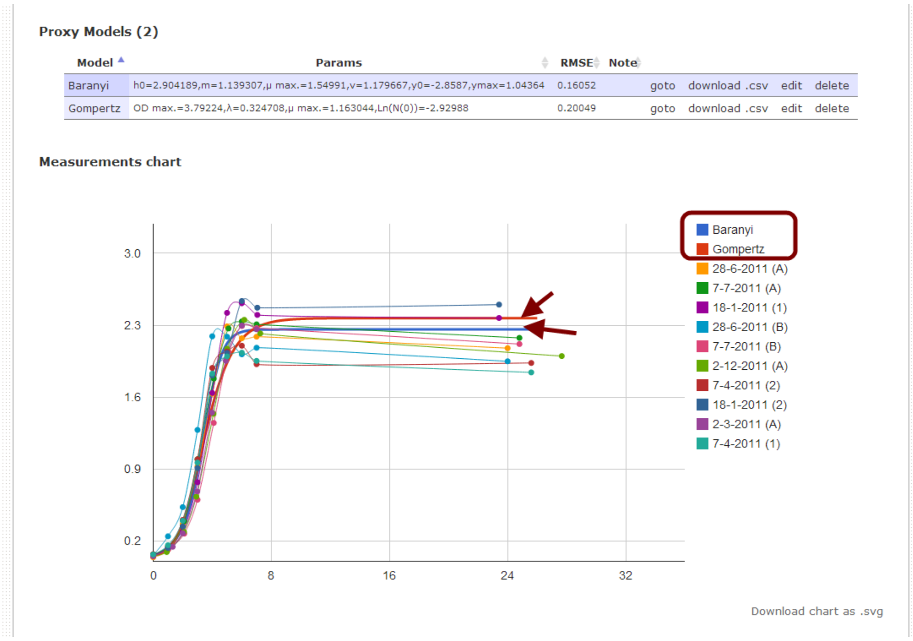

4.8.2 Batch estimation

Batch estimation allows to perform multiple estimation in parallel, testing different parameter's range for datasets in different projects / experiments.

This is suited to perform simultaneous parameter estimation to a large dataset (using a fixed parameter range) or to recalculate results using the last known good parameter range.

1. Navigate to a model's detail page

2. Click on "Estimate in batch"

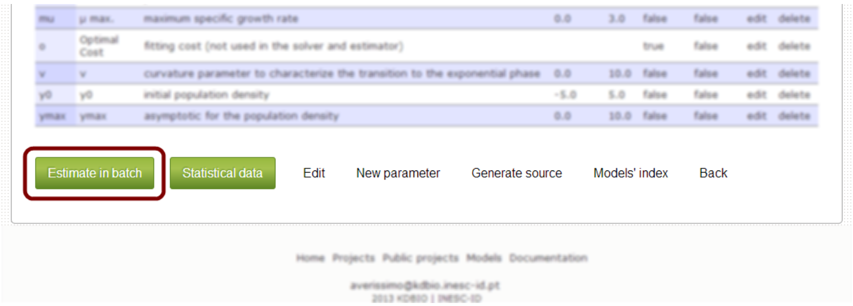

3. Select the parameter range

   Either the last known parameter range used for the parameter estimation (for each measurement), or a fixed range.

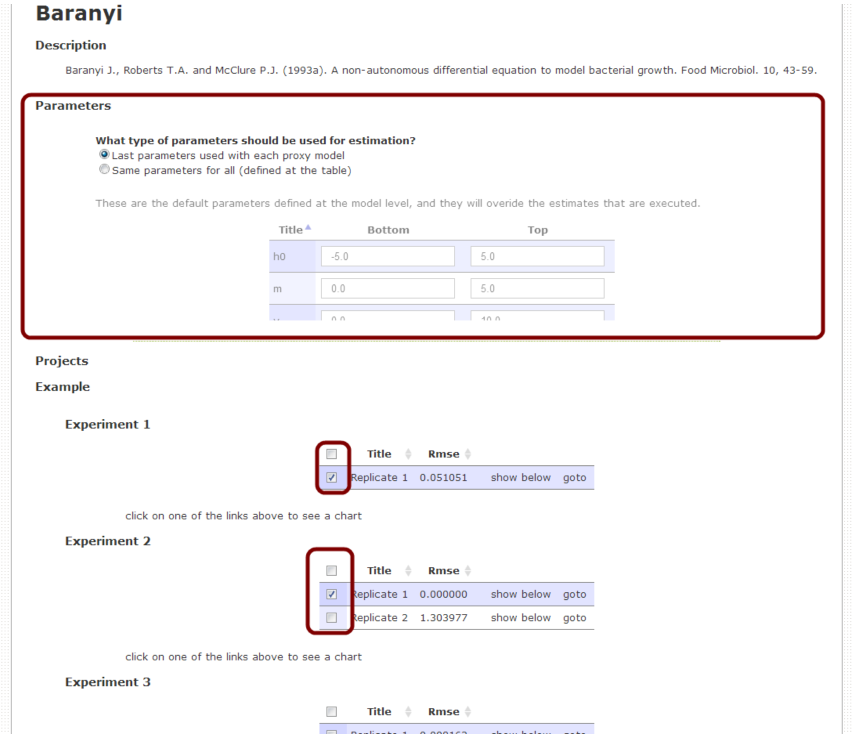

4. Click on "Estimate paramenters" to start

As this operation uses a background process to perform all the parameter estimation, a note is added to the proxy model. When the results are calculated the note is removed and the parameter values are shown.

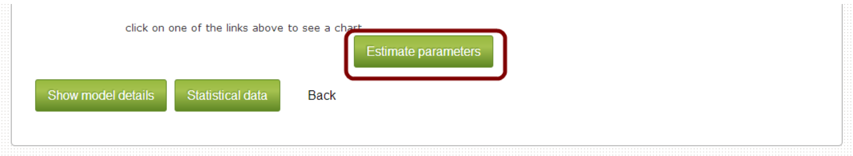

4.9 Generate a new Model

There are two existing methods to create a new model to use in the application.

1. The first is described in 5.7;
2. The second can be done through the BGFit application itself, generating the necessary source files.

4.9.1 Create a new Model

1. Navigate to the model index by clicking on the Models menu

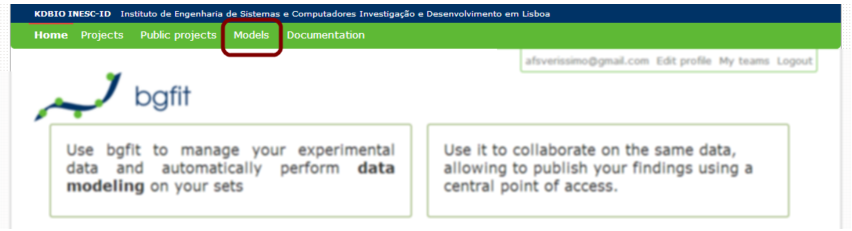

2. Click on New model button

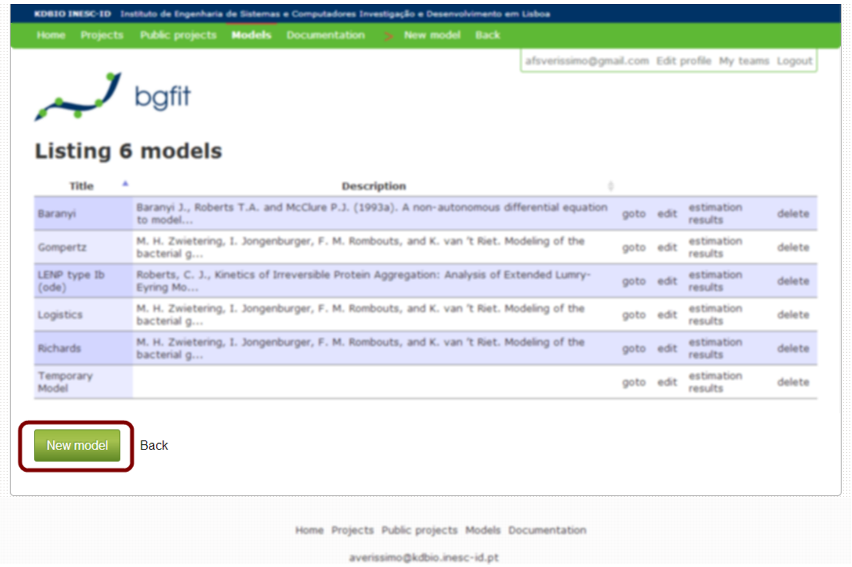

3. Introduce the necessary informatiion The user needs to introduce the necessary information to define the model. A model can be defined by:
   (a) An already published model that has a simulation and estimation interface (i.e. two urls that indicate these interfaces)
   (b) The equation that defines it.

note: for both cases it is necessary to define the parameters (either on the creation page or later in the models page)

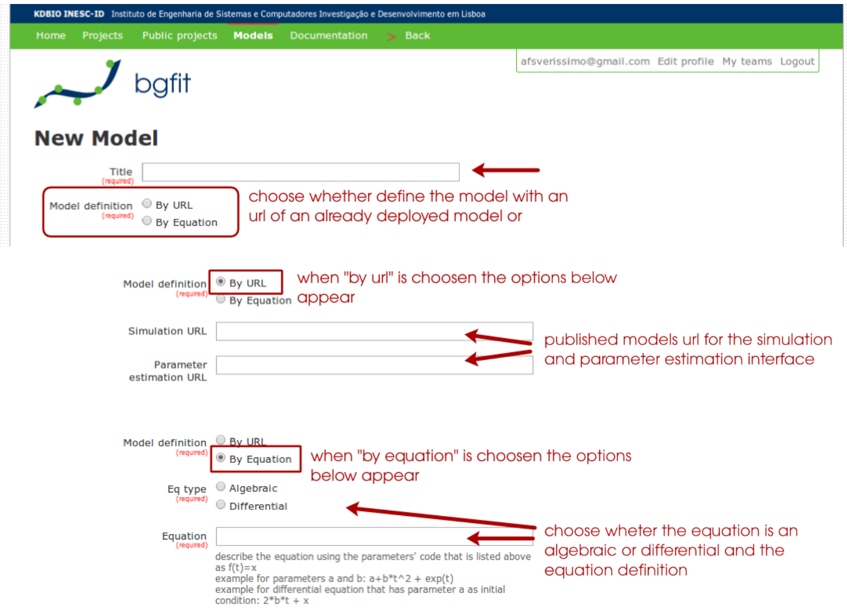

Add parameters and the necessary parameters and other options. To add a parameter the user needs to click on + add new parameter and define the parameters detail.

More information on this on section 2.2.

note: the code defines the parameter as described in the equation, it should start with an letter and cannot contain spaces or special characters (think of a matlab variable).

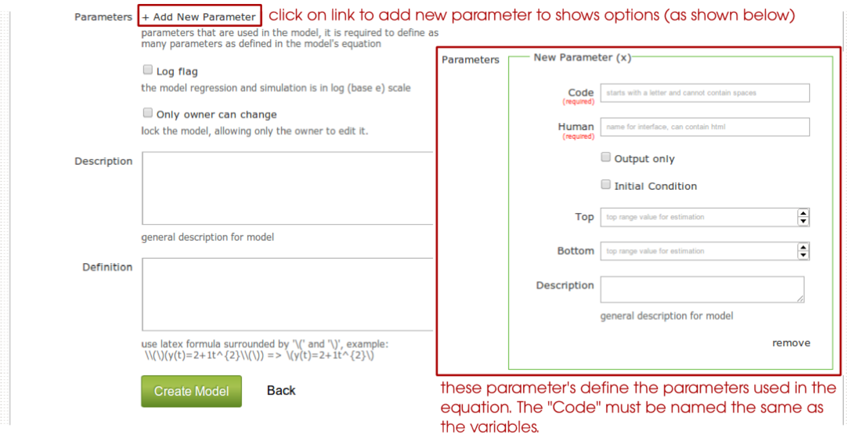

4.9.2 Add models parameters (if added later on the details page)

Click on the New parameter button and add the models necessary parameters

(a) Click on New parameter

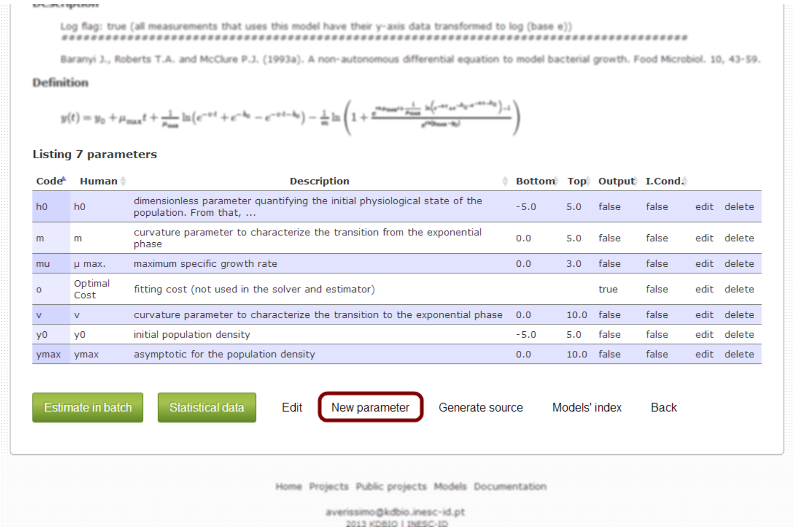

(b) Introduce the necessary information:

- Code: the variable name that will uniquely identify the parameter;
- Human: the human friendly symbol or name for the parameter;
- Output only: If true this parameter will not be passed to the model, but will be determined as a result of a model call;
- Initial condition: If true this will be used as an initial condition for differential models.

4.9.3 Generate source files

The equation can be latter defined or corrected.

4. Click on "Generate source" on the model's detail page

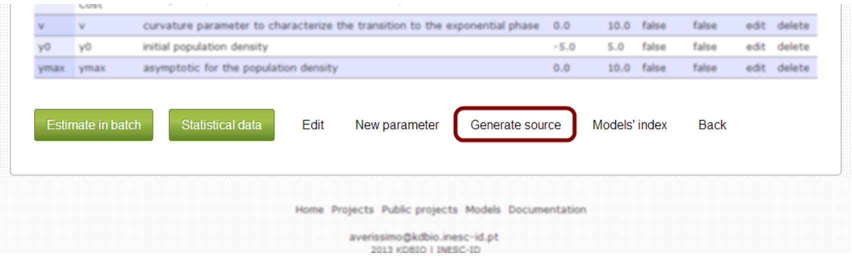

5. Select the equation
   - Algebraic;
   - Differential.

6. Describe the equation

   Introduce the right hand side of the equation. Such as $f(t) = a + b^2 exp(t)$ with parameters a and b:

   ```
   a + b * t^2 + exp(t)
   ```

   example for differential equation that has parameter a (as initial condition) and b:

   ```
   2 * b * t + x
   ```

7. Save the model

   Click on "Update model"

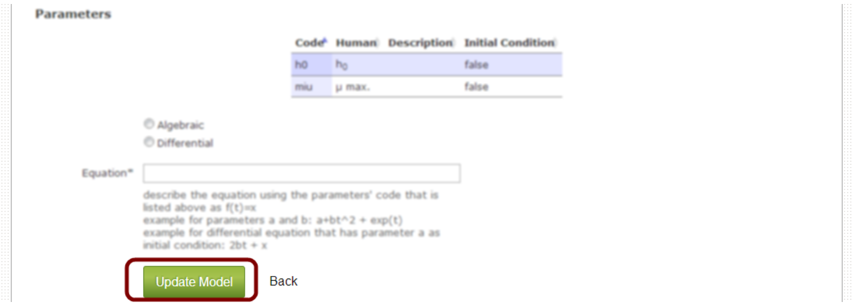

4.9.4 Download source files

The generated files are then accessible as links in the page (model, estimator and simulator functions)

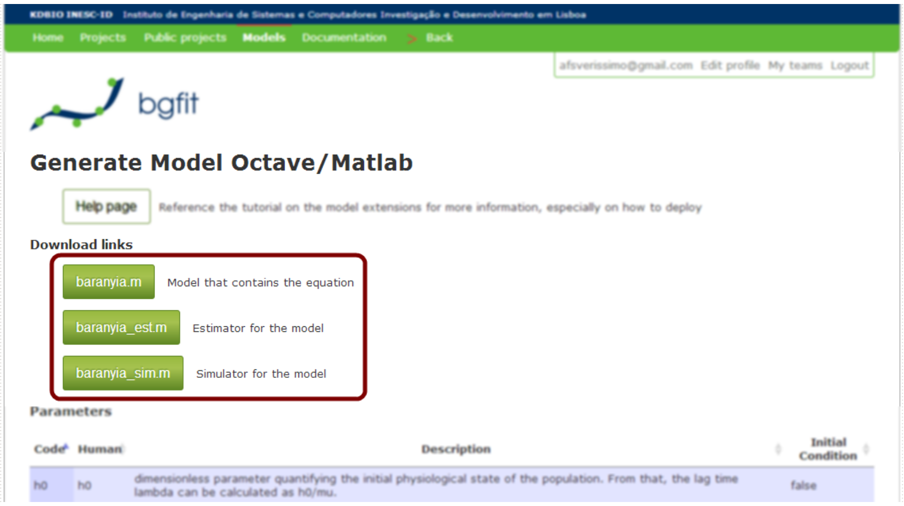
Home
Projects
Public projects
Models
Documentation
Back
Edit profile
My teams
Logout
bgfit
Generate Model Octave/Matlab
Help page
Reference the tutorial on the model extensions for more information, especially on how to deploy
Download links
baranyia.m
Model that contains the equation
baranyia_est.m
Estimator for the model
baranyia_sim.m
Simulator for the model
Parameters
Code
Human
Description
Initial Condition
h0
h0
dimensionless parameter quantifying the initial physiological state of the population. From that, the lag time lambda can be calculated as h0/mu.
false

4.10 Manual Regression

A manual regression allows to calculate a linear regression on the data using a subset of points from the measurement. This calculation is performed on a logarithm scale.

1. Navigate to the Measurement's detail page

   To start the user should navigate to the measurement's detail page from an existing project.

2. Click on "Manual regression"

   To perform a manual linear regression on measurement data the user should navigate to a measurement's detail page

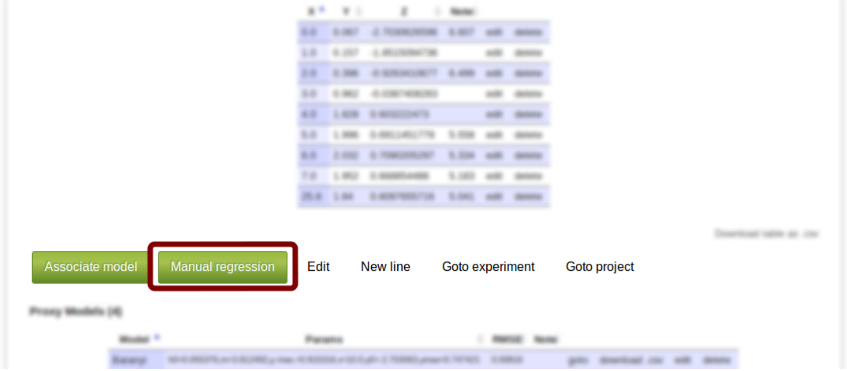

3. Select points

   The user should select two or more points from the data table.

   To select points the user should use the mouse and click on the points that will be used in the linear regression.

   In order to select multiple points the user should click on two (or more) different entries on the table by either:

   (a) Press the "shift" key to select sequential points;
   (b) Press the "ctrl" key to select specific points.

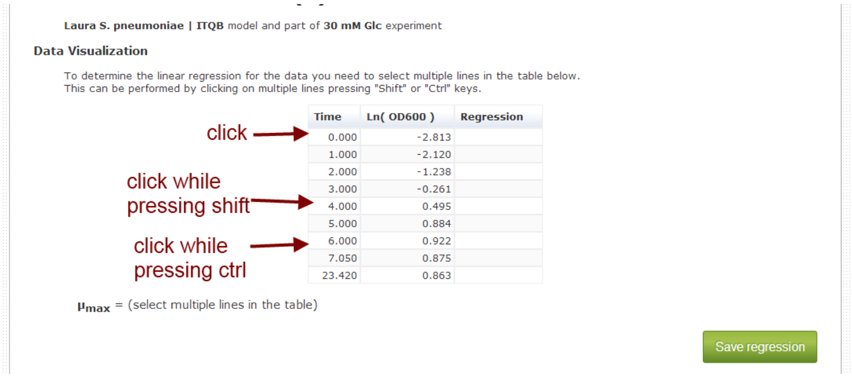

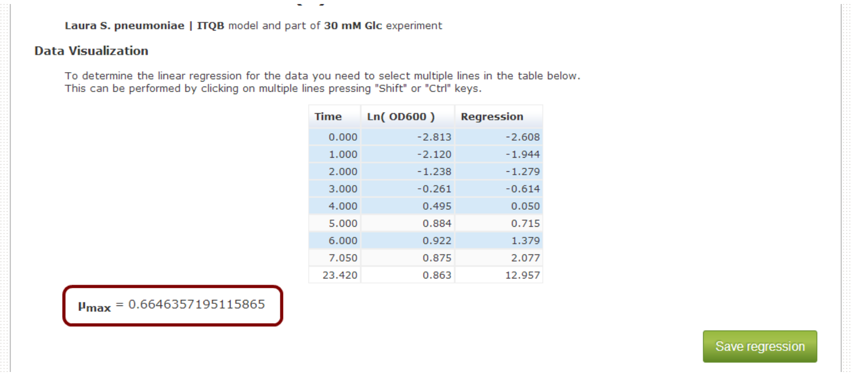

4. Live preview

   The chart below the table shows a live preview of the linear regression, as well as the max for the regression.

   $$y = a + \mu_{max} \cdot x$$

   The preview is updated as the selected points change.

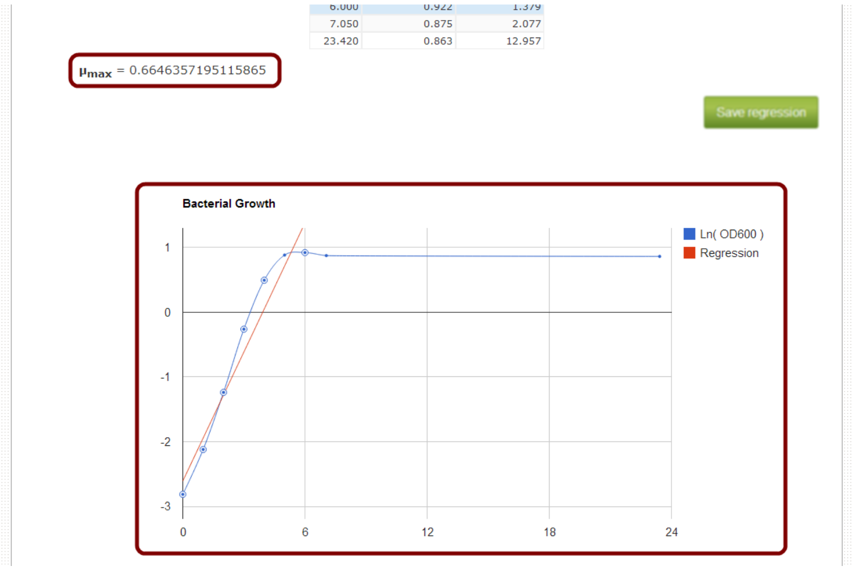

5. Save the regression

   Click on "Save regression" button

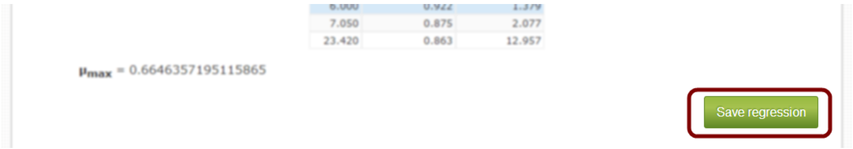

5 Modeling Extensions

5.1 Overview

This is a Octave/Matlab package that provides methods for non-linear parameter estimation for a REST application. It reads a web query string and returns a JSON file with the results.

5.2 Download

Model extensions can be downloaded at https://github.com/averissimo/model_blackbox

5.3 Technical Overview

This package source code is divided in:

- Models in *models* directory that separates
- Algebraic models in *models/algebraic*
- Differential models (ODE) in *models/differential*
- In each sub-directory a different model is defined by 3 different functions: *model / estimator / simulator*
- Auxiliary functions in the *toolbox* directory that contains:
- Auxiliary functions in the *toolbox/src* directory
- SBTOOLBOX2 models structure that uses *toolbox/estimators*, *toolbox/models*, and *toolbox/simulators* directories
- Compile model script that pre-compiles the SBTOOLBOX2 models as MEX executables

5.4 Models

Model Extensions currently implements dynamic models that can implement algebraic or differential (ODE) equations.

The package already has some implemented models that are described in literature:

- **Baranyi** – *Baranyi J., Roberts T.A. and McClure P.J. (1993a). A non-autonomous differential equation to model bacterial growth. Food Microbiol. 10, 43-59;*
- **Gompertz, Logistics, Richards and Schnute** – *M. H. Zwietering, I. Jongenburger, F. M. Rombouts, and K. van t Riet. Modeling of the bacterial growth curve. Applied and Environmental Microbiology, 56(6):18751881, June 1990;*

- **LENP type Ib** – *Roberts, C. J., Kinetics of Irreversible Protein Aggregation: Analysis of Extended LumryEyring Models and Implications for Predicting Protein Shelf Life. J. Phys. Chem. B 2003, 107 (5), 1194-1207;*
- **Live Cell Growth** – *Milotti et al, Interplay between distribution of live cells and growth dynamics of solid tumours, Sci Rep., 2, December 2012.*

As an example, the "Baranyi" algebraic model (baranyia) will use the following equation, described as F(t) with auxiliary function F2(t)

```
F2(t) = ( 1 ./ mu ) .* log( exp(-v .* t) + exp(-h0)
- exp(-v .* t - h0) );

F(t) = y0 + mu .* t + F2 - (1 ./ m) *
log( 1 + ( exp( m .* mu .* t + F2) - 1 ) ./ exp(m .* (ymax -y0) ) );
```

For a differential model, such as the "LENP type Ib", a differential equation, such as:

```
dxdt = -2 .* fr_ .* fr_ .* k11_ .* ( n_ / 2 ) .* x .* x;
```

note: x = x and only the right side of the equation should be included in the definition.

5.5 Requirements

This Octave/Matlab package is a blackbox application for parameter estimation and model simulation.
It supports three different backends:

- Octave (optim package)
- Matlab (optimization toolbox)
- Matlab (SBTOOLBOX2 toolbox)

We reccomend to use either Octave or Matlab own toolboxes, as the SBTOOLBOX2 might become unstable if the data scale is increased.
The models in the models base directory are compatible with both Octave and Matlab.

5.5.1 Requirements for Octave-based models

- Octave environment (tested with 3.6.2)
- Optim package octave.sourceforge.net/optim

5.5.2 Requirements for Matlab-based models

- Matlab environment
- Optimization toolbox
- Compiler toolbox

5.5.3 Requirements for SBTOOLBOX2-based models

- Matlab environment
- Compiler toolbox
- SBTOOLBOX2 toolbox sbtoolbox2.org
- SBPD toolbox sbtoolbox2.org

5.6 Structure

The usage of any model is dependant of having three files:

- model: where the model's equation is defined
- estimator : .m file that defines the necessary steps to estimate parameters
- simulator : .m file that simulates a curve with given parameters

These files allow to generate a cgi script for the model that can be accessed online or in a local computer

5.7 Create a new model blackbox

5.7.1 Octave / Matlab model

1. Clone the repository;
2. Navigate to the models folder;
3. Navigate to the algebraic or differential folder, depending on the model type. If it is defined as an algebraic equation or as an differential, choose the right folder;
4. Copy the TEMPLATE folder and name it to the model name;
5. Open each of the .m files and change it accordingly;
    - model: write the equation, if it is a differential equation don't forget the initial condition;
    - estimator: change the 'model' variable to the name of the model;
    - simulator: change how the parameters are set in alphabetical order and the 'model' variable.

5.7.2 Compile for octave

Navigate to the base dir and run:

```
make octave
```

5.7.3 Compile for matlab

Navigate to the base dir add to the Makefile file a target using any of the existing as a template:

```
make %model_name%
```

5.7.4 Compile for SBTOOLBOX2 model

1. Create a SBTOOLBOX2 model (SBModel) and copy it the directory:

   ```
   source/models/
   ```

2. compile the model calling the compile_model.m function, ex:

   ```
   compile_model('models/baranyi')
   ```

3. copy the following files to the same directory and name it after the model, preserving the suffix (just to help organizing the files' function

   ```
   source/estimators/TEMPLATE_est.m
   ```

   ```
   source/simulators/TEMPLATE_sim.m
   ```

4. change the source code to reflect the name of the model by replacing all the occurences of Gompertz to "Yourmodel"

   ```
   IMPORTANT: the first letter must be Uppercase
   ```

5. in the simulators/Yourmodel_sim.m you must change the code to handle the model's parameters

6. add the makefile target following the existing templates

7. run

   ```
   make yourmodel_est yourmodel_sim clean
   ```

5.8 Test the model

You can simulate the query by calling in Octave/Matlab the respective function with the arguments: * simulate flag: if 1 then it will use te test data defined at the top of the function * draw plot: draws a plot of the results

5.9 Deploy

These functions can be deployed as cgi scripts throught a REST API and return a JSON response. The parameters can be passed in the url or as POST.

The main requirement is a web server sucha as Apache or Nginx

5.9.1 Octave

To deploy a model using Octave, it is only required to have Octave installed (tested with 3.6.1) and have the toolbox on path.

It is necessary to desactivate octave verbose output. When using a linux server we reccomend the use of a shebang script (see the "bin" folder)

```
#! /bin/sh
exec octave --silent --no-window-system $@
```

The package allows to generate all the cgi scripts by running the following command in the root directory:

```
make octave
```

This will create a estimator and a simulator cgi scripts for all models inside the "models" directory.

See Requirements for Octave-based models above

5.9.2 Matlab or SBTOOLBOX2

The process to deploy Matlab models (both Matlab or SBTOOLBOX2) is a bit different, as the user needs to compile the model as a standalone application before.

For this the Compiler Toolbox is required to generate executable files. There are already makefile targets for existing models that can be used as templates.

As an example, to deploy the baranyi algebraic models as Matlab cgi scripts, the following command should be issued:

```
make baranyia
```

The makefile will generate a standalone that only needs the Matlab libraries to run.

See Requirements for Matlab-based models above for matlab models, or the Requirements for SBTOOLBOX2-based models for SBTOOLBOX2 models

6 Questions and Sugestions

Contact the team at svinga@kdbio.inesc-id.pt
